# Effect of CO_2_ Partial Pressure on the Corrosion Behavior of J55 Carbon Steel in 30% Crude Oil/Brine Mixture

**DOI:** 10.3390/ma11091765

**Published:** 2018-09-18

**Authors:** Haitao Bai, Yongqing Wang, Yun Ma, Qingbo Zhang, Ningsheng Zhang

**Affiliations:** 1 Institute of Petroleum and Gas Engineering, Southwest Petroleum University, Chengdu 610500, China; baihaitao_xsyu@126.com; 2 College of Petroleum Engineering, Key Laboratory of Environment Pollution Control Technology of Oil Gas and Reservoir Protection in Shaanxi Protection, Xi’an Shiyou University, Xi’an 710065, China; mayun_xian123@126.com (Y.M.); renpeng_xian@126.com (Q.Z.); zhangnsh_xian@126.com (N.Z.)

**Keywords:** mechanism of CO_2_ corrosion, J55 carbon steel, CO_2_ partial pressure, localized corrosion

## Abstract

The influence of CO_2_ partial pressure on the corrosion properties, including corrosion rate, morphology, chemical composition, and corrosion depth, of J55 carbon steel in 30% crude oil/brine at 65 °C was investigated. A corrosion mechanism was then proposed based on the understanding of the formation of localized corrosion. Results showed that localized corrosion occurred in 30% crude oil/brine with CO_2_. The corrosion rate sharply increased as the CO_2_ partial pressure (P${\mathrm{co}}_{2}$) was increased from 0 to 1.5 MPa, decreased from P${\mathrm{co}}_{2}$ = 1.5 MPa to P${\mathrm{co}}_{2}$ = 5.0 MPa, increased again at P${\mathrm{co}}_{2}$ = 5.0 MPa, and then reached a constant value after P${\mathrm{co}}_{2}$ = 9.0 MPa. The system pH initially decreased, rapidly increased, and then stabilized as CO_2_ partial pressure was increased. In the initial period, the surface of J55 carbon steel in the CO_2_/30% crude oil/brine mixtures showed intense corrosion. In conclusion, CO_2_ partial pressure affects the protection performance of FeCO_3_ by changing the formation of corrosion scale and further affecting the corrosion rate.

## 1. Introduction

In recent years, the carbon dioxide flooding enhanced oil recovery (CO_2_-EOR) technology has been widely applied worldwide [1,2,3] and has made a positive contribution to the geological reserves of carbon. However, CO_2_-EOR is expected to significantly increase the corrosion failure risk of tubes [4,5]. The acceptable rate of wellbore corrosion in China is less than 0.076 mm·year^−1^ [6], and the qualitative categorization of carbon steel corrosion rates for oil production systems in the US includes low (<0.025 mm·year^−1^), moderate (0.025–0.12 mm·year^−1^), high (0.13–0.25 mm·year^−1^), and severe (>0.25 mm·year^−1^) [7]. When water cut is greater than 50%, the corrosion rates of carbon steel (API 5CT L80) and P110 steel are 3.4–34.2 and 0.03–5.0 mm·year^−1^, respectively [8,9], which are far beyond the acceptable range. Thus, many studies have focused on CO_2_ corrosion, especially on the effect of environment on corrosion.

Mass loss during CO_2_ corrosion is generally related to environmental conditions, such as temperature, pressure, salt concentration, solution pH, and CO_2_ partial pressure. CO_2_ partial pressure and protective scale considerably impact corrosion rate. Many studies demonstrated that the CO_2_ corrosion rate of carbon steel increases with increasing CO_2_ pressure [10,11,12]. The concentration of H_2_CO_3_ increases as CO_2_ partial pressure increases, which accelerates the cathodic reactions and increases the corrosion rate [13,14,15,16]. CO_2_ partial pressure affects the protective properties and components of the corrosion product layer by changing the system pH. Other studies [15,16,17,18,19,20,21] indicated that FeCO_3_ is the main composition in the corrosion product layer that is formed on corroded carbon steel surface exposed to CO_2_ environment. A.H. Mustafa [17] reported that the corrosion product film of X52 steel is inhomogeneous and porous in CO_2_/formation water at different CO_2_ pressures (10, 40, and 60 bar) and 60 °C, and the corrosion product layer is mainly composed of FeCO_3_ and Fe_3_C. However, increasing CO_2_ partial pressure does not often accelerate corrosion. Yoon-Seok Choi [19] proposed that the corrosion rates of carbon steel measured in CO_2_-saturated water show no significant difference (19.5–20.1 mm·year^−1^) with pressure (4, 6, and 8 MPa) at 50 °C. Preliminary studies mainly focused on the influences of CO_2_ partial pressure on corrosion in brine environment and of water cut on corrosion in CO_2_/crude oil/brine environment, but few studies focused on the influence of CO_2_ partial pressure on corrosion in crude oil/brine environment. Thus, understanding the effect of CO_2_ partial pressure on the corrosion behavior of J55 carbon steel in crude oil/brine mixtures is important.

In the present work, the effect of CO_2_ partial pressure on the corrosion behavior of J55 carbon steel was compared in CO_2_/30% crude oil (*v*/*v*, the same below)/brine mixtures. The corrosion rates were determined by weight mass loss, and the maximum corrosion depth was obtained with an optical digital microscope. The morphology and composition of the formed corrosion product film were characterized by scanning electron microscopy (SEM), energy dispersive spectrometry (EDS), and X-Ray diffraction (XRD).

## 2. Materials and Methods

The material used in this work was J55 carbon steel with a composition (wt.%) of 0.36% C, 0.30% Si, 1.45% Mn, 0.016% P, 0.004% S, 0.051% Cr, 0.009% Ni, 0.07% Cu, and Fe balance. The specimen for weight loss test was machined into 50 mm × 10 mm × 3 mm and a hole of 6 mm with an exposed area of 13.6 cm^2^. The samples were placed in acetone to remove oil on the surface and then immersed in ethanol for 5 min for further degreasing and dehydration. The samples were dried in cold air, packed with filter paper, and then placed in the dryer for 4–7 h. Finally, the size and weight of the samples were measured to within an accuracy of 0.1 mg.

The corrosive medium is a mixture of oil and water, the crude oil is obtained from the C_8_ reservoir of a certain block in Changqing oilfield, and the brine is the simulated solution prepared according to the composition of the brine in the reservoir. The compositions of the crude oil and simulated solution are shown in Table 1 and Table 2, respectively.

**Figure 1:**
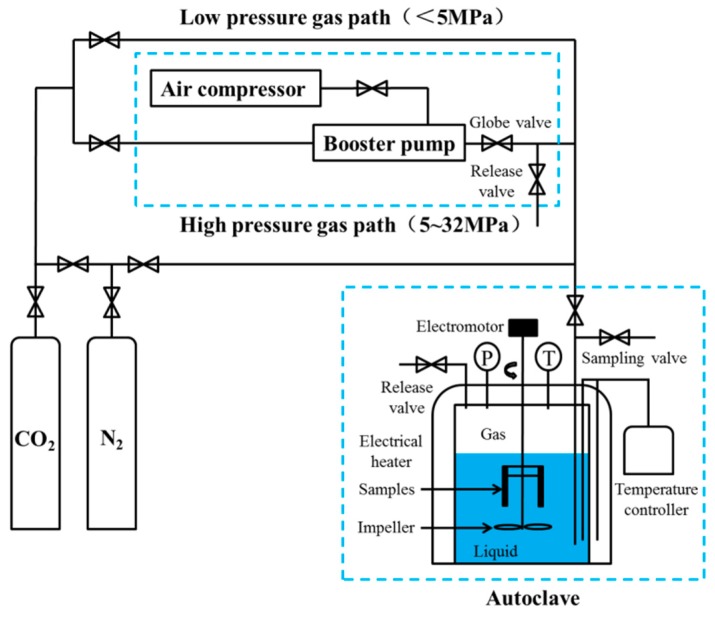
Flow chart of steel corrosion rate evaluation system (Mass loss method).

In CO_2_-EOR, gas channelling often occurs [22,23], during which the CO_2_ partial pressure rises from the bottom hole to no more than 15 MPa in the C_8_ reservoir. Corrosion test was carried out in the PARR-4578 autoclave (Parr Instrument Company, Champaign, IL, USA) by using the weight-loss method, and the schematic is shown in Figure 1. A 1 L aliquot of the mixture of 30% crude oil/brine was added to the autoclave, and the dissolved oxygen was purged in the solution with a small amount of nitrogen gas for 4 h under a pressure of 0.5 MPa [13] and a temperature of 65 °C. The autoclave was pressured with pure N_2_ gas to the experimental values (total pressure value—CO_2_ partial pressure) and with CO_2_ gas to a total pressure value of 15 MPa for 2 days at the running speed of 0.5 m·s^−1^ (200 r·min^−1^).

After corrosion induction, the three corroded samples were divided into two groups for scanning electron microscope (SEM), energy dispersive spectrometer (EDS), and X-ray diffraction (XRD) analyses of the corrosion scales formed on the steel surface. After these tests, the three corroded samples were subjected to mass loss tests to determine the average corrosion rate.

The corrosion rate of the steel was determined by the mass loss technique in accordance with the ASTM (American Society for Testing Materials) G1-03-Standard practice for preparing, cleaning, and evaluating corrosion [24]. Immediately after corrosion induction, the samples were rinsed with distilled water and the crude oil on the surface was removed with acetone. Corrosion products were removed with an ultrasonic cleaner. Then, the samples were immersed in an acid cleaning solution (500 mL of HCl and 3.5 g of hexamethylenamine diluted with water to 1000 mL) for 10 min, and the corrosion products on the surface were removed. After being immersed, the samples were thoroughly washed with distilled water until the acid cleaning solution on the surface was completely removed. Then, the samples were placed in ethanol for cleaning and dehydration twice. The samples were dried in cold air, packed with filter paper, and then placed in the dryer for 4–7 h. Finally, the samples were weighed to within an accuracy of 0.1 mg. The corrosion rate was calculated as follows:

(1) $$r_{\mathrm{corr}}=\frac{8.76\times 10^{4}\times (m-m_{t})}{S\times t\times \rho }$$

where *r_corr_* is the average corrosion rate, mm·year^−1^; *m* is the weight of the test sheet before the experiment, g; *m_t_* is the weight of the test sheet after the experiment, g; *S* is the whole surface contacted with solution, cm^2^; *ρ* is the density of tested steel, g·cm^−3^, which is 7.86 g·cm^−3^ in the case of carbon steel; and *t* is the immersion duration, h. The mean corrosion rate error was calculated using three parallel specimens in each test.

The surface microstructure of the corrosion product scales on the surface of corroded samples was analyzed via SEM (FEI Quanta 600F microscope, FEI Corporation, Hillsboro, TX, USA). The elemental compositions of the corrosion product scales were estimated by EDS (OXFORD INCA energy 350, Oxford Instrument, Oxford, UK). The composition of the corroded samples was performed with XRD (Bruker D8 XRD, Bruker Corporation, Karlsruhe, Germany).

**Figure 2:**
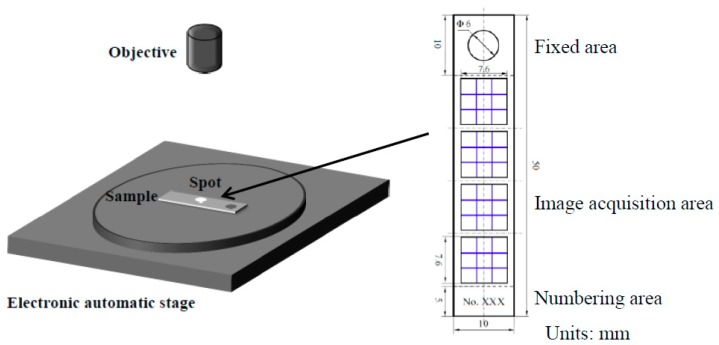
Schematic of image acquisition.

The maximum corrosion depth of the corroded samples was analyzed with an optical digital microscope (OLYMPUS DSX500, Olympus Corporation, Tokyo, Japan) after removal of the corrosion product layers by using the acid cleaning solution. Under bright-field mode, the corroded sample surface was subjected to grand horizon three dimensions (3D) image capture using adjacent visual synthetic diagram mode. The magnification was 100 times, with a 3 × 3 nine-image synthetic diagram and an overlap ratio of 10%. Four points on the front and back surfaces of the samples were collected, as shown in Figure 2. The area of the 3 × 3 nine-image synthetic diagram was 7612 μm × 7612 μm, the total area of image acquisition was 4.63 cm^2^, and 43.27% of the exposed surface area was occupied, which was much larger than that in other studies [15,16,17,18,19,20,21]. The maximum corrosion depth could be acquired by comparing the corrosion depth measured in different areas. Therefore, the method can also accurately reflect the maximum corrosion depth of the corroded samples.

## 3. Results

### 3.1. Weight Loss Tests

**Figure 3:**
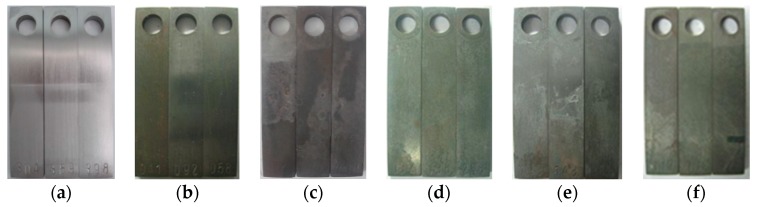
Macroscopic morphologies of J55 carbon steel before corrosion test: (**a**) before corrosion test and after the removal of corrosion scales under different CO_2_ partial pressures; (**b**) P${\mathrm{co}}_{2}$ = 0 MPa; (**c**) P${\mathrm{co}}_{2}$ = 1.5 MPa; (**d**) P${\mathrm{co}}_{2}$ = 5.0 MPa; (**e**) P${\mathrm{co}}_{2}$ = 9.0 MPa; and (**f**) P${\mathrm{co}}_{2}$ = 15.0 MPa.

**Figure 4:**
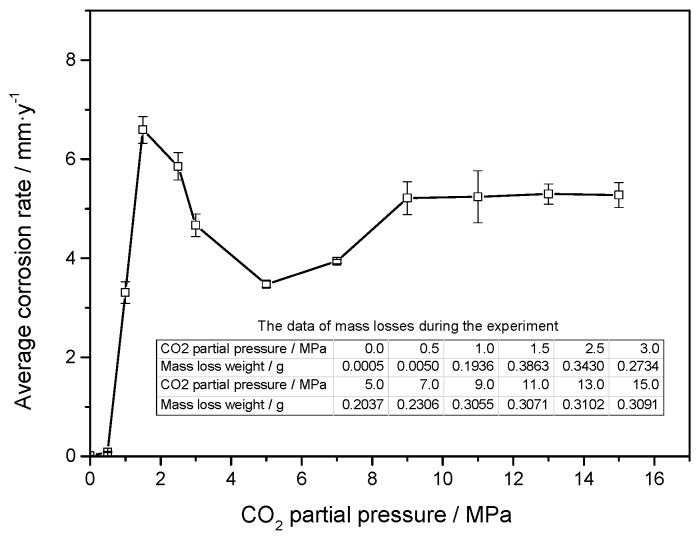
Average corrosion rate determined by mass loss technique as a function of CO_2_ partial pressure for steel immersed in 30% crude oil/brine mixtures.

Figure 3 shows the macroscopic morphologies of the J55 carbon steel before corrosion test and after the removal of corrosion scales under different CO_2_ partial pressures. Localized corrosion occurred on the surface of the J55 carbon steel. As shown in Figure 4, the average corrosion rate of the J55 carbon steel after immersing in a CO_2_/crude oil/brine environment initially increased and then decreased with increasing CO_2_ partial pressure before finally stabilizing. When the CO_2_ partial pressure was increased from 0 to 1.5 MPa, the corrosion rate of J55 increased sharply. The concentration of H_2_CO_3_ increased as the partial pressure of CO_2_ was increased, which decreased the system pH and therefore increased the corrosion rate [13,14,15,16]. When the CO_2_ partial pressure was increased from 1.5 MPa to 5.0 MPa, the corrosion rate of J55 decreased. With the continuous increase in CO_2_ partial pressure, a protective layer gradually formed on the surface of the J55 carbon steel. When the CO_2_ partial pressure was increased from 5.0 to 9.0 MPa, the corrosion rate of J55 increased. The protective layer formed on the surface of J55 may be dissolved gradually, thereby increasing the corrosion rate [14,15]. When the CO_2_ partial pressure was increased from 9.0 MPa to 15.0 MPa, the corrosion rate of J55 was almost constant. A protective layer formed faster on the steel surface as the CO_2_ partial pressure was increased [17,18,19]. When CO_2_ dissolved in water equilibrium, CO_2_ solubility almost no longer increased with increasing CO_2_ partial pressure. Thus, the system pH was almost invariable [25], and the protective layer was not dissolved.

### 3.2. Microstructure and Composition of the Corrosion Scale

**Figure 5:**
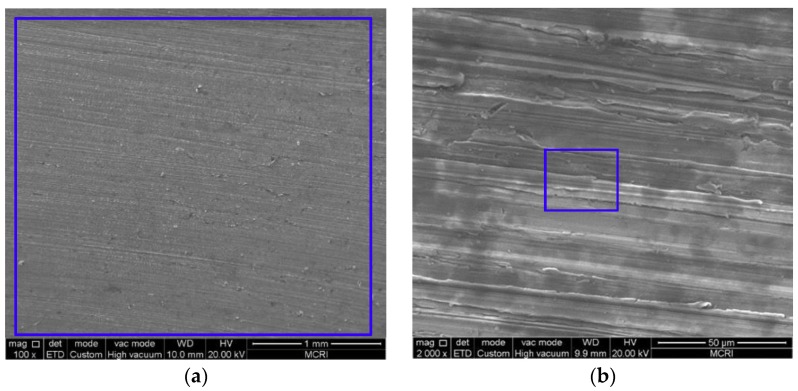
SEM images of corrosion scales formed on the steel surface in 30% crude oil/brine mixtures at P${\mathrm{co}}_{2}$ = 0 MPa: (**a**) ×100; (**b**) ×2000.

**Figure 6:**
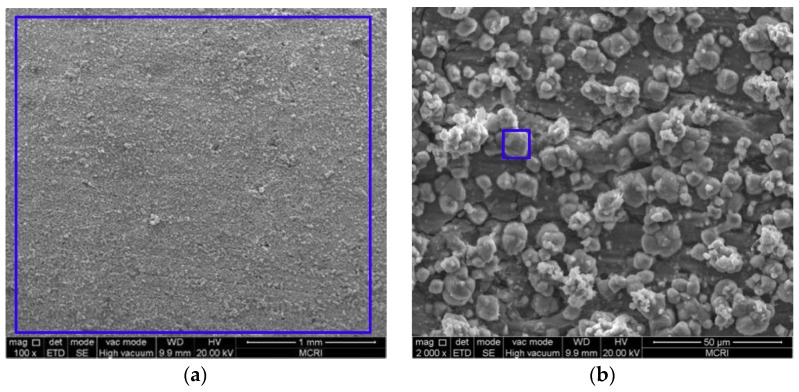
SEM images of the corrosion scales formed on the steel surface in 30% crude oil/brine mixtures at P${\mathrm{co}}_{2}$ = 1.5 MPa: (**a**) ×100; (**b**) ×2000.

**Figure 7:**
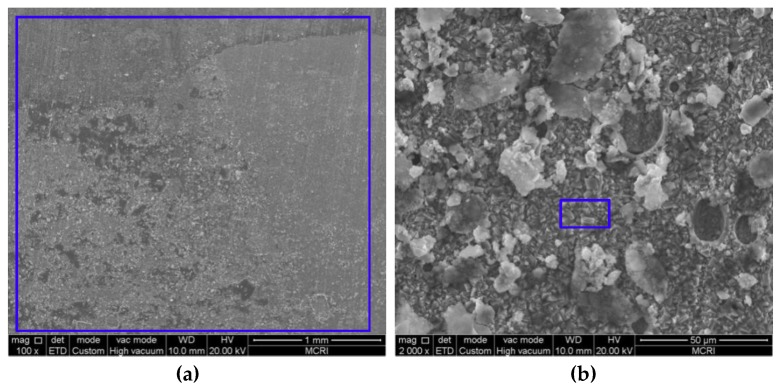
SEM images of the corrosion scales formed on the steel surface in 30% crude oil/brine mixtures at P${\mathrm{co}}_{2}$ = 5.0 MPa: (**a**) ×100; (**b**) ×2000.

**Figure 8:**
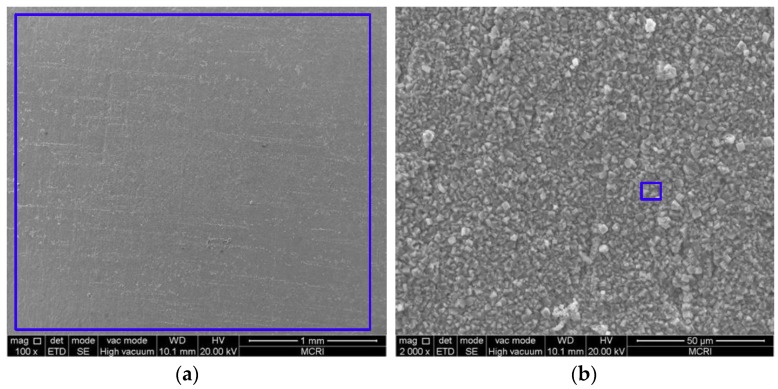
SEM images of the corrosion scales formed on the steel surface in 30% crude oil/brine mixtures at P${\mathrm{co}}_{2}$ = 9.0 MPa: (**a**) ×100; (**b**) ×2000.

**Figure 9:**
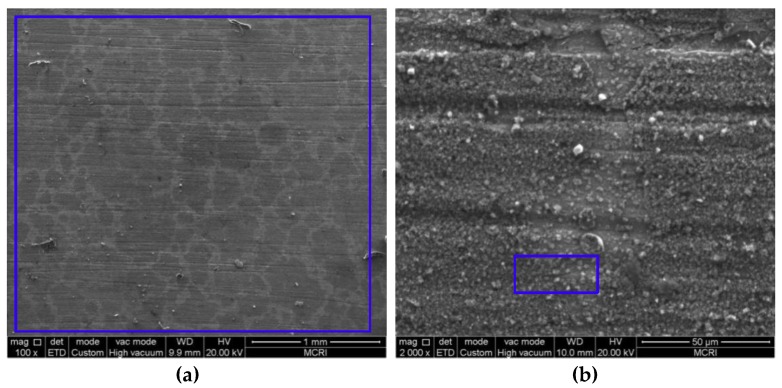
SEM images of corrosion scales formed on the steel surface in 30% crude oil/brine mixtures at P${\mathrm{co}}_{2}$ = 15.0 MPa: (**a**) ×100; (**b**) ×2000.

Figure 5, Figure 6, Figure 7, Figure 8 and Figure 9 show the SEM images of the corrosion scales formed on the J55 steel surface as a function of CO_2_ partial pressure in 30% crude oil/brine mixtures at the same magnification (×100 or ×2000). EDS was performed on the corrosion product scales of the tested samples. Table 3 shows the EDS spectra of the corrosion scale in the inner surface of the blue line region in Figure 4, Figure 5, Figure 6, Figure 7 and Figure 8, respectively. Figure 5 shows the SEM images of the corrosion scales formed on the J55 steel surface at P${\mathrm{co}}_{2}$ = 0 MPa and 65 °C. The polishing marks were still visible on the surface of the J55 steel, and no visible signs of corrosion were observed on the sample. The corrosion product mainly consisted of Fe_3_C (the content ratio of Fe and C atoms is about 1:3) and minor constituents of alloying elements from the carbon steel matrix. Figure 6 shows the SEM images of the corrosion scales formed on the J55 steel surface at P${\mathrm{co}}_{2}\;$ = 1.5 MPa and 65 °C. The surface was severely attacked and showed disperse FeCO_3_ and CaCO_3_ scales and minor constituents of alloying elements from the carbon steel matrix. Figure 7 shows the SEM images of the corrosion scales formed on the J55 steel surface at P${\mathrm{co}}_{2}$ = 5.0 MPa and 65 °C. A large part of the surface was attacked and fully covered by FeCO_3_ and CaCO_3_. Figure 8 and Figure 9 show the SEM images of the corrosion scales formed on the J55 steel surface at P${\mathrm{co}}_{2}$ = 9.0 and 15 MPa. The surface was almost covered by the protective FeCO_3_ layer and few CaCO_3_. At P${\mathrm{co}}_{2}$ = 15.0 MPa, the corrosion product layer was thicker and denser than that at P${\mathrm{co}}_{2}$ = 9.0 MPa.

The main elements of the corrosion products in the 30% crude oil/brine environment without CO_2_ were Fe and C, and the content ratio of iron and carbon atoms was about 1:3, indicating that the corrosion products consisted mainly of FeC_3_. The main elements of the corrosion products in the CO_2_/30% crude oil/brine environment were O, Fe, and Ca, indicating that the corrosion products consisted mainly of FeC_3_ and mixed carbonate (Fe_x_Ca_1−x_CO_3_) [26,27]. At P${\mathrm{co}}_{2}$ = 1.5 and 5.0 MPa, the minor constituents of alloying elements from the carbon steel were detected, indicating that the surface was not fully covered by the corrosion product layer. At P${\mathrm{co}}_{2}$ = 9.0 and 15.0 MPa, the minor constituents of alloying elements from the carbon steel were not detected, suggesting that the surface was fully covered by the corrosion product layer.

**Figure 10:**
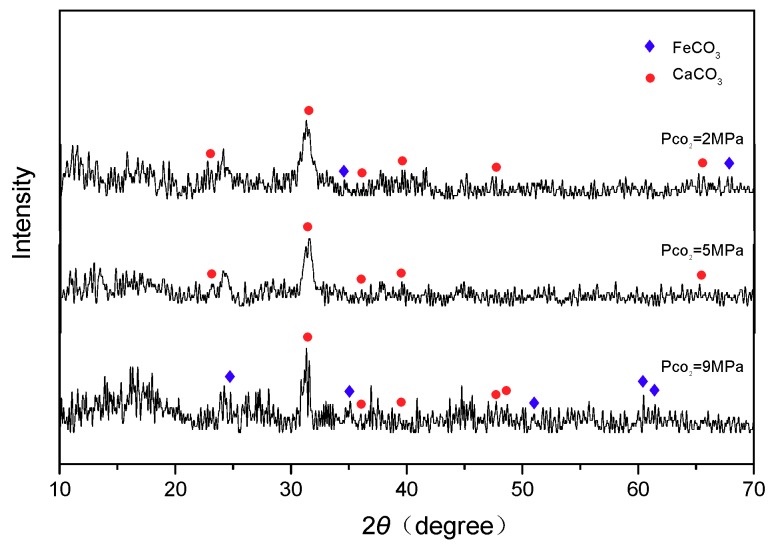
XRD spectra of surface layer on the corroded samples.

Figure 10 shows the XRD spectra of the surface layer on the corroded samples immersed in CO_2_/30% crude oil/brine mixtures. Related research [15,16,17,18,19,20,21] reported that the main CO_2_ corrosion product of carbon steel is FeCO_3_. The compositions of the corrosion product layer in the CO_2_/30% crude oil/brine mixtures were similar and mainly consisted of the complex salt of CaCO_3_ and FeCO_3_. This result may be attributed to the presence of metal cation isomorphous substitution in CO_2_ corrosion [27]. When the [Fe^2+^] × [CO_3_^2−^] in the medium exceeds FeCO_3_ solubility product K_sp_ (FeCO_3_), that is, when the FeCO_3_ supersaturation in the medium is $S=\left\{\left[{\mathrm{Fe}}^{2+}\right]\times \left[{\mathrm{CO}}_{3}^{2-}\right]\right\}/\left\{K_{\mathrm{sp}}\left({\mathrm{FeCO}}_{3}\right)\right\}>1$, the FeCO_3_ would be deposited on the metal surface. As shown in the following form [27]:

(2) $${\mathrm{Fe}}^{2+}{+\mathrm{CO}}_{3}^{2-}\to {\mathrm{FeCO}}_{3}\;(s)$$

The Ca^2+^ in the solution to the replacement in FeCO_3_ crystal Fe^2+^ and the formation of the Fe (Ca) CO_3_ complex can be expressed as

(3) $${\mathrm{Ca}}^{2+}+{\mathrm{FeCO}}_{3}\;(s)\to {\mathrm{Fe}}^{2+}+{\mathrm{CaCO}}_{3}\;(s)$$

### 3.3. Maximum Corrosion Depth Tests

**Figure 11:**
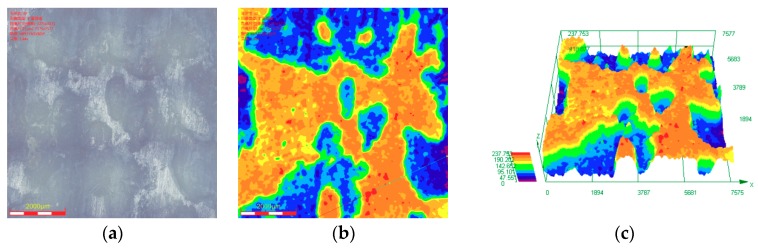
Corrosion depth analysis on cleaned surface of the sample exposed to 30% crude oil/brine condition at P${\mathrm{co}}_{2}$ = 1.0 MPa and 65 °C: (**a**) corrosion morphology; (**b**) corrosion depth distribution contour diagram; and (**c**) corrosion depth distribution 3D diagram.

**Figure 12:**
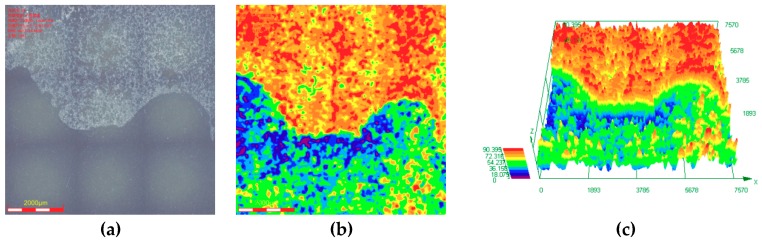
Corrosion depth analysis on the cleaned surface of the sample exposed to 30% crude oil/brine condition at P${\mathrm{co}}_{2}$ = 1.5 MPa and 65 °C: (**a**) corrosion morphology; (**b**) corrosion depth distribution contour diagram; and (**c**) corrosion depth distribution 3D diagram.

**Figure 13:**
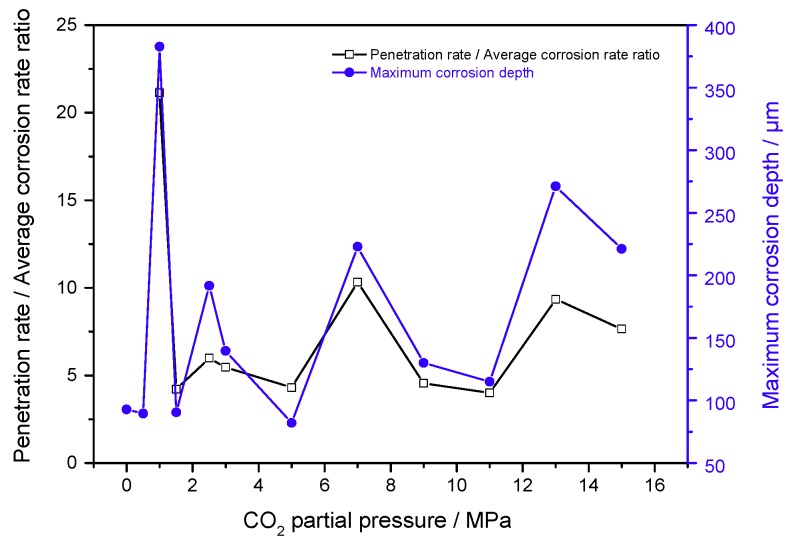
Maximum corrosion depth and penetration rate/average corrosion rate ratio of J55 carbon steel surface as a function of CO_2_ partial pressure in 30% crude oil/brine mixtures.

Figure 11 shows that the maximum corrosion depth of the cleaned sample surface exposed to 30% crude oil/brine condition at P${\mathrm{co}}_{2}$ = 1.0 MPa and 65 °C was 237.753 μm. The maximum corrosion depths measured in the seven other regions were compared, and the maximum corrosion depth of the cleaned sample surface exposed to 30% crude oil/brine condition at P${\mathrm{co}}_{2}$ = 1.0 MPa and 65 °C was 382.742 μm. As shown in Figure 12, the maximum corrosion depth of the cleaned sample surface exposed to 30% crude oil/brine condition at P${\mathrm{co}}_{2}$ = 1.5 MPa and 65 °C, where the average corrosion rate was the highest, was 90.395 μm. The type of corrosion damage changed from localized corrosion to mesa corrosion. Figure 13 shows the maximum corrosion depth and penetration rate/average corrosion rate ratio of the J55 carbon steel surface after removal of the corrosion product layers by using acid cleaning solution as a function of CO_2_ partial pressure in the 30% crude oil/brine mixtures. The maximum corrosion depth varied with the increase in CO_2_ partial pressure possibly because of the protection conferred by the corrosion product layer. The variation trend of the penetration rate/average corrosion rate ratio of the J55 carbon steel surface was the same as that of the maximum corrosion depth as the CO_2_ partial pressure was increased. The penetration rate/average corrosion rate ratios were greater than 4, indicating that local corrosion occurred on the surface of the carbon steel [8,18]. At P${\mathrm{co}}_{2}$ = 1.0 MPa, the corrosion depth was the largest at 382.742 μm, which corresponded to 69.8504 mm/a. This penetration rate was considerably greater than the weight-loss corrosion rate (3.3058 mm·year^−1^) shown in Figure 3, thereby confirming local attack.

## 4. Discussion

### 4.1. Variation of pH with CO_2_ Partial Pressure

As the CO_2_ partial pressure was increased, the corrosion rate of the J55 carbon steel initially increased, decreased, increased again, and then stabilized. This result is different from the report of some scholars that the CO_2_ corrosion rate of carbon steel increases with increasing CO_2_ pressure [13,15,28]. G.A. Zhang [13] reported the corrosion rate of N80 carbon steel increases from 19.13 mm·year^−1^ to 23.91 mm·year^−1^ when the CO_2_ partial pressure increases from 5 MPa to 8 MPa. When the samples were immersed in formation water for 96 h at 60 °C and a rotational speed of 2 m·s^−^^1^. Zhang Y. The authors of [15] proposed that the corrosion rate of X65 carbon steel increases from 1.64 mm·year^−1^ to 7.26 mm·year^−1^ when the samples are immersed in aqueous environment for 168 h at 80 °C and 1–9.5 MPa. M. Seiersten [28] reported that the corrosion rates of X65 carbon steels in aqueous CO_2_ conditions range within 1–6 mm·year^−1^ at 40 °C and 7.5–9 MPa. The corrosion rate in the literature is greater than that in the test. The difference may be attributed to the different tested corrosion media and the obvious corrosion inhibition effect of crude oil that can greatly reduce the corrosion rate of CO_2_ [9].

The acid value of crude oil is 0.107 mg KOH·g^−1^, and crude oil would not substantially change the system pH. The initial system pH is 6.5, and the change in pH is mainly caused by the dissolution of CO_2_ in water. CO_2_ dissolves in water and forms carbonic acid in situ, and the acidity of the solution increases, thereby decreasing the pH. The equilibrium relationship can be described this process:

(4) $$H_{2}O⇌H^{+}+{\mathrm{OH}}^{-},\;K_{w}$$

(5) $${\mathrm{CO}}_{2(\mathrm{aq})}+H_{2}O⇌H^{+}+{\mathrm{HCO}}_{3}{}^{-},\;K_{a1}$$

(6) $${\mathrm{HCO}}_{3}{}^{-}⇌H^{+}+{\mathrm{CO}}_{3}{}^{2-},\;K_{a2}$$

(7) $$K_{w}=x_{H^{+}}x_{{\mathrm{OH}}^{-}}(\gamma_{H^{+}}\gamma_{{\mathrm{OH}}^{-}}),$$

(8) $$K_{a1}=\frac{x_{H^{+}}x_{{\mathrm{HCO}}_{3}{}^{-}}(\gamma_{H^{+}}\gamma_{{\mathrm{HCO}}_{3}{}^{-}})}{x_{{\mathrm{CO}}_{2(\mathrm{aq})}}\gamma_{{\mathrm{CO}}_{2(\mathrm{aq})}}},$$

(9) $$K_{a2}=\frac{x_{H^{+}}x_{{\mathrm{CO}}_{3}{}^{2-}}(\gamma_{H^{+}}\gamma_{{\mathrm{CO}}_{3}{}^{2-}})}{x_{{\mathrm{HCO}}_{3}{}^{-}}\gamma_{{\mathrm{HCO}}_{3}{}^{-}}},$$

where *K_w_* is the ionization constant of water, *K*_*a*1_ is the first ionization constant of CO_2_, *K*_*a*2_ is the secondary ionization constant of CO_2_, *x* is the concentration of subscript ion, mol·L^−1^, and *γ* is the activity coefficient of subscript ion. 

Stumm and Morgon [29] calculated the first and secondary ionization equilibrium constant of CO_2_ in water, and Morshall and Franch [30] calculated the ionization equilibrium constant of water.

(10) $$\log\left(K_{a1}\right)=\frac{134737.5}{T}-2211.492-0.30004T+785.768\log T-\frac{9036500}{T^{2}},$$

(11) $$\log\left(K_{a2}\right)=\frac{24784.1}{T}-452.824-0.078515T+162.47\log T-\frac{1713800}{T^{2}},$$

(12) $$\log\left(K_{w}\right)=-4.098-\frac{3245.2}{T}+\frac{223620}{T^{2}}-\frac{39840000}{T^{3}}+(13.956-\frac{1262.3}{T}+\frac{856410}{T^{2}})\log(\rho ),$$

where *T* is the temperature of the system, K; *P* is the partial pressure of CO_2_ in the system, bar; and *ρ* is the density of the system, g·cm^−3^.

Wiebe et al. [31,32] calculated the solubility of CO_2_ in water at 12–100 °C, and assumed that the solubility of CO_2_ in water was not related to the concentration of solution and pH value. Ziegler [33] fitted the solubility data of Wiebe to the following empirical formula:

(13) $$S_{{\mathrm{CO}}_{2}}=4.11-\frac{4329.91}{T}+\frac{9.20\times 10^{5}}{T^{2}}-2.5\times 10^{-3}P+3.75\frac{P}{T}-951.30\frac{P}{T^{2}}-1.52\times 10^{-7}P^{2}+0.34\ln(P)$$

where $S_{{\mathrm{CO}}_{2}}$ is the total concentration of CO_2_ dissolved in water, mol·kg^−1^.

The first ionization of the carbonated solution is the main reaction, and *K*_*a*1_ is much larger than *K_w_* and *K*_*a*2_. When estimating the pH of the CO_2_–H_2_O system, the ionization of water and the secondary ionization of carbonic acid can be neglected. Carbonic acid is a weak acid, and its $x_{{\mathrm{CO}}_{2}}$ is much larger than its $x_{H^{+}}$. In carbonate solution, except for CO_2(aq)_, the concentration of other ions is negligible. Carbonic acid is a dilute solution with an ionic activity coefficient of about 1. The density of carbonic acid is similar to that of pure water, and the density of pure water slightly varies with temperature, then $x_{H^{+}}$ ≈ $S_{{\mathrm{CO}}_{2}}$. In this way, $x_{H^{+}}$ and pH are calculated as follows:

(14) $$x_{H^{+}}=\sqrt{K_{a1}\times x_{{\mathrm{CO}}_{2(\mathrm{aq})}}}=\sqrt{K_{a1}\times S_{{\mathrm{CO}}_{2}}},$$

(15) $$\mathrm{pH}=-\log\left(x_{H^{+}}\times \gamma_{H^{+}}\right)=-\log\left(x_{H^{+}}\right)-\log\left(\gamma_{H^{+}}\right)=-\log\left(x_{H^{+}}\right).$$

**Figure 14:**
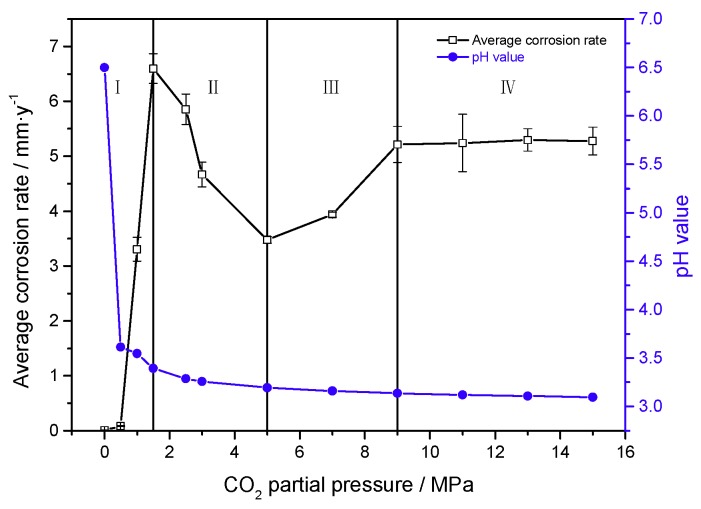
Average corrosion rate and estimated pH value as a function of CO_2_ partial pressure in 30% crude oil/brine mixtures.

Figure 14 shows the average corrosion rate and estimated pH value as a function of CO_2_ partial pressure in 30% crude oil/brine mixtures. The system pH decreased with increasing CO_2_ partial pressure. When the CO_2_ partial pressure was small, the system pH decreased significantly with increasing CO_2_ partial pressure. When the CO_2_ partial pressure was high, the pH of the system decreased insignificantly with increasing CO_2_ partial pressure. The dissolution of CO_2_ in water to achieve equilibrium continued to increase the CO_2_ partial pressure but the pH value almost no longer increased [25]. Therefore, the different average corrosion rates in 30% crude oil/brine with CO_2_ partial pressure were not only caused by pH changes but by a series of chemical changes.

### 4.2. Formation Mechanism of Localized Corrosion

**Figure 15:**
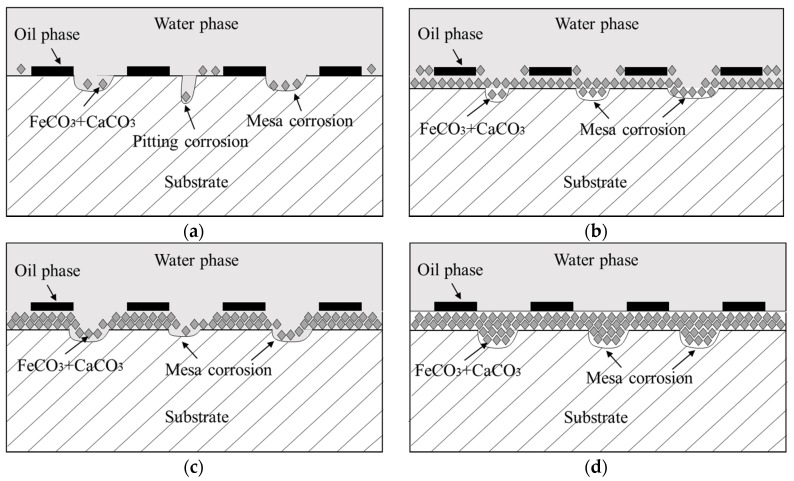
Schematic models for the localized corrosion of J55 steel in the different CO_2_/30% crude oil/brine mixtures: (**a**) model I: generating scattered corrosion scale (P${\mathrm{co}}_{2}$ = 0.5–1.5 MPa); (**b**) model II: gradually generating protective corrosion scale (P${\mathrm{co}}_{2}$ = 1.5–5.0 MPa); (**c**) model III: dissolving protective corrosion scale (P${\mathrm{co}}_{2}$ = 5.0–9.0 MPa); and (**d**) model IV: generating protective corrosion scale (P${\mathrm{co}}_{2}$ = 9.0–15.0 MPa).

With the change in CO_2_ partial pressure, the corrosion rate of the J55 carbon steel changed significantly in 30% crude oil/brine mixtures, which indicated that the corrosion mechanism also changed. Figure 13 shows the partition graph of the corrosion rate in CO_2_/30% crude oil/brine mixtures as the change in CO_2_ partial pressure. Water-in-oil and oil-in-water emulsions coexist in 30% crude oil/brine mixtures, and the ratio of oil-in-water emulsion is large. Crude oil and water can all moisten the metal surface. The crude oil with corrosion inhibition was not evenly adsorbed on the metal surface, causing localized corrosion. As shown in Figure 14, corrosion models were proposed to clarify the influence of CO_2_ partial pressure on the mechanism of localized corrosion. The four stages describing the formation of localized corrosion are as follows:

Model I (shown in Figure 15a): At P${\mathrm{co}}_{2}$ = 0–1.5 MPa, the corrosion rate increased rapidly with the increase in CO_2_ partial pressure. When the CO_2_ partial pressure was small, the system pH decreased significantly with increasing CO_2_ partial pressure, similar to the estimated pH value shown in Figure 14. The concentration of H_2_CO_3_ increased with increasing CO_2_ partial pressure, which accelerated the cathodic reactions, increased the corrosion rate [13,14,15,16], and finally increased the Fe^2+^ content. The corrosion scale precipitated and the scattered corrosion scale appeared on the surface, but the solubility products of FeCO_3_ and CaCO_3_ increased because the pH decreased, as shown in Figure 6b. Pitting may also occur locally due to the presence of crude oil and Cl^−^ [9].

Model II (shown in Figure 15b): At P${\mathrm{co}}_{2}$ = 1.5–5.0 MPa, the corrosion rate decreased with increasing CO_2_ partial pressure. At the initial stage of the experiment, the metal surface suffered strong localized corrosion. This result is consistent with the conclusions of many researchers [5,13,14,15,16]. The Fe^2+^ concentration increased and acidic concentration decreased rapidly on the steel surface. The nucleation and growth of FeCO_3_ typically start on the steel surface where the pH and FeCO_3_ saturation values are the highest [34]. The FeCO_3_ layer restricted the transport of H^+^ in and Fe^2+^ out; thus, the corrosion rate decreased with the increase in CO_2_ partial pressure. The reduction of the system pH value also dissolved the corrosion scale, but the dissolution rate of the corrosion product layer was lower than the precipitation rate as the CO_2_ partial pressure was increased.

Model III (shown in Figure 15c): At P${\mathrm{co}}_{2}$ = 5.0–9.0 MPa CO_2_, the corrosion rate increased with increasing CO_2_ partial pressure, which is in good agreement with the literature [28]. With the increase in CO_2_ partial pressure from 5 to 9.0 MPa, CO_2_ phase changed to a supercritical state, and the solubility of CO_2_ in crude oil increased rapidly [35]. When the decrease in pH value promoted the dissolution of protective layers, CO_2_ possibly transferred from crude oil to the aqueous phase, supplemented with consumed H^+^ dissolving the product layer [36]. Thus, the surface of carbon steel was exposed to corrosive medium, and corrosion reaction was promoted [13,16]. The dissolution rate of the corrosion product layer was greater than the precipitation rate as the CO_2_ partial pressure was increased.

Model IV (shown in Figure 15d): At P${\mathrm{co}}_{2}$ = 9.0~15.0 MPa, the corrosion rate was almost constant with the increase in CO_2_ partial pressure. This result can be attributed to the almost-constant system pH value (about 3.10–3.14), as shown in Figure 14. At the initial stage of the experiment, the metal surface suffered strong localized corrosion. The production and dissolution of corrosion scale were carried out simultaneously. Finally, a dense, complete, and protective corrosion product layer formed rapidly on the metal surface. Yoon-Seok Choi et al. [18] also obtained similar results, i.e., the corrosion rates of L80 in 25 wt.% NaCl solution started out high but ended up being very low at 90 °C and 12 MPa CO_2_ pressure. Pitting may occur under dense protective layer, as shown in Figure 13.

## 5. Conclusions

Based on the observed corrosion behavior of J55 carbon steel in different CO_2_/30% crude oil/brine mixtures at 65 °C, we conclude the following:

(1) The corrosion rate sharply increased as the CO_2_ partial pressure was increased from 0 to 1.5 MPa, decreased from P${\mathrm{co}}_{2}$ = 1.5 MPa to P${\mathrm{co}}_{2}$ = 5.0 MPa, increased again at P${\mathrm{co}}_{2}$ = 5.0 MPa, and then reached a constant value after P${\mathrm{co}}_{2}$ = 9.0 MPa. 

(2) In 30% crude oil/brine mixtures, the surface of J55 carbon steel was covered by FeCO_3_ and CaCO_3_. The surface of the J55 carbon steel suffered localized corrosion in different CO_2_/30% crude oil/brine mixtures at 65 °C.

(3) The system pH initially decreased, rapidly increased, and then stabilized as CO_2_ partial pressure was increased. The CO_2_ partial pressure changed the system pH and CO_2_ solubility in crude oil, which further affected the formation and protection performance of the corrosion product layer.

## Figures and Tables

**Table 1:** Composition properties of crude oil.

Property	Unit	Value
Kinematic viscosity (65 °C)	mm^2^·s^−1^	7.254
Acid value	mg KOH·g^−1^	0.107
Sulfur content	wt.%	0.08
Wax content	wt.%	12.86
Colloid	wt.%	2.31
Asphaltene	wt.%	0.60

**Table 2:** Properties of simulated brine preparation.

Property	Unit	Value
NaCl	g·L^−1^	18.5028
CaCl_2_	g·L^−1^	13.7338
MgCl_2_	g·L^−1^	0.5897
Na_2_SO_4_	g·L^−1^	0.2440
NaHCO_3_	g·L^−1^	0.0631
salinity	g·L^−1^	33.0000

**Table 3:** EDS of the corrosion scale of immersion in 30% crude oil/brine mixtures under different CO_2_ partial pressures.

Element (At. %)	$P{\mathrm{co}}_{2}=0\;\mathrm{MPa}$		$P{\mathrm{co}}_{2}=1.5\;\mathrm{MPa}$		$P{\mathrm{co}}_{2}=5.0\;\mathrm{MPa}$		$P{\mathrm{co}}_{2}=9.0\;\mathrm{MPa}$		$P{\mathrm{co}}_{2}=15.0\;\mathrm{MPa}$	
	Whole	Local	Whole	Local	Whole	Local	Whole	Local	Whole	Local
C K	75.70	76.23	37.34	33.40	50.29	33.25	27.87	18.66	63.00	62.49
O K	0.34	1.50	44.87	55.80	36.73	45.10	46.48	53.98	23.68	25.52
Si K	/	/	/	/	0.21	/	/	/	/	/
Cr K	/	/	0.29	/	/	/	/	/	/	/
Ca K	/	/	3.49	3.17	/	0.20	1.84	2.29	0.44	0.59
Cl K	0.14	/	0.37	0.16	0.30	0.37	/	/	/	/
Mn K	0.40	0.30	0.54	0.35	0.14	0.28	/	/	/	/
Fe K	23.42	21.97	13.10	7.12	12.33	20.80	23.81	25.07	12.88	11.40
total	100.0	100.0	100.0	100.0	100.0	100.0	100.0	100.0	100.0	100.0

## References

[1] Uddin M., Jafari A., Perkins E. (2013). Effects of mechanical dispersion on CO_2_ storage in Weyburn CO_2_-EOR field-numerical history match and prediction. Int. J. Greenh. Gas. Control.

[2] Barnes D., Harrison B., Grammer G.M., Asmus J. (2013). CO_2_-EOR and geological carbon storage resource potential in the Niagaran pinnacle reef trend, lower Michigan, USA. Energy Procedia.

[3] Choi J.W., Nicot J.P., Hosseini S.A., Clift S.J., Hovorka S.D. (2013). CO_2_ recycling accounting and EOR operation scheduling to assist in storage capacity assessment at a U.S. gulf coast depleted reservoir. Int. J. Greenh. Gas. Control.

[4] Wang Z.J., Cates M.E., Langan R.T. (1998). Seismic monitoring of a CO_2_ flood in a carbonate reservoir: A rock physics study. Geophysics.

[5] Choi Y.S., Nesic S. (2010). Effect of impurities on the corrosion behavior of CO_2_ transmission pipeline steel in supercritical CO_2_-water environments. Environ. Sci Technol..

[6] (2012). Water Quality Standard and Practice for Analysis of Oilfield Injecting Waters in Clastic Reservoirs.

[7] (2005). Preparation, Installation, Analysis, and Interpretation of Corrosion Coupons in Oilfield Operations.

[8] Farelas F., Choi Y.S., Nesic S. (2013). Corrosion behavior of deep water oil production tubing material under supercritical CO_2_ environment: Part 2-effect of crude oil and flow. Corrosion.

[9] Sun J.B., Sun C., Zhang G.A., Zhao W.B., Wang Y. (2016). Effect of water cut on the localized corrosion behavior of P110 tube steel in supercritical CO_2_/oil/water environment. Corrosion.

[10] Wei L., Pang X.L., Gao K.W. (2015). Effects of crude oil on the corrosion behavior of pipeline steel under wet CO_2_ conditions. Mater. Perform..

[11] Efird K.D., Jasinski R.J. (1989). Effect of the crude oil on corrosion of steel in crude oil/brine production. Corrosion.

[12] Lin G., Bai Z., Zhao X. (2006). Effect of temperature and pressure on the morphology of carbon dioxide corrosion scales. Corrosion.

[13] Zhang G.A., Liu D., Li Y.Z., Guo X.P. (2017). Corrosion behavior of N80 carbon steel in formation water under dynamic supercritical CO_2_ condition. Corros. Sci..

[14] Zhang Y.C., Pang X.L., Qu S.P., Li X., Gao K.W. (2011). The relationship between fracture toughness of CO_2_ corrosion scale and corrosion rate of X65 pipeline steel under supercritical CO_2_ condition. Int. J. Greenh. Gas. Control.

[15] Zhang Y.C., Pang X.L., Qu S.P., Li X., Gao K.W. (2012). Discussion of the CO_2_ corrosion mechanism between low partial pressure and supercritical condition. Corros. Sci..

[16] Cui Z.D., Wu S.L., Li C.F., Zhu S.L., Yang X.J. (2004). Corrosion behavior of oil tube steels under conditions of multiphase flow saturated with super-critical carbon dioxide. Mater. Lett..

[17] Mustafa A.H., Angeles C.B, Ismail M.C. (2013). Inhibition of CO_2_ corrosion of X52 steel by imidazoline-based inhibitor in high pressure CO_2_ -water environment. J. Mater. Eng. Perform..

[18] Choi Y.S., Farelas F., Neši S., Magalhães A.A.O., Andrade C.D.A. (2014). Corrosion behavior of deep water oil production tubing material under supercritical CO_2_ environment: Part 1-effect of pressure and temperature. Corrosion.

[19] Choi Y.S., Nešić S. (2011). Determining the corrosive potential of co transport pipeline in high pco–water environments. Int. J. Greenh. Gas. Control.

[20] Nesic S. (2012). Effects of multiphase flow on internal CO_2_ corrosion of mild steel pipelines. Energy Fuels.

[21] Nesic S., Lunde L. (1994). Carbon dioxide corrosion of carbon steel in two-phase flow. Corrosion.

[22] Gozalpour F., Ren S.R., Tohidi B. (2005). CO_2_ EOR and storage in oil reservoir. Oil Gas Sci. Technol..

[23] Hao H., Hou J., Zhao F., Song Z., Hou L., Wang Z. (2016). Gas channeling control during CO_2_ immiscible flooding in 3D radial flow model with complex fractures and heterogeneity. J. Pet. Sci. Eng..

[24] (1999). Standard Practice for Preparing, Cleaning, and Evaluating Corrosion Test Specimens.

[25] Roosen C., Ansorge S.M., Mang T., Leitner W., Greiner L. (2007). Gaining pH-control in water/carbon dioxide biphasic systems. Green Chem..

[26] Esmaeely S.N., Choi Y.S., Young D., Nesic S. (2013). Effect of calcium on the formation and protectiveness of iron carbonate layer in CO_2_ corrosion. Corrosion.

[27] Esmaeely S.N., Young D., Brown B.N., Nesic S. (2017). Effect of incorporation of calcium into Iron carbonate protective layers in CO_2_ corrosion of mild steel. Corrosion.

[28] Seiersten M. Material selection for separation, transportation and disposal of CO_2_ corrosion. Proceedings of the Corrosion Conference.

[29] Stumm W., Morgan J.J. (1996). Aquatic chemistry: Chemical equilibria and rates in natural waters. J. Chem. Educ..

[30] Marshall W.L., Franck E.U. (1981). Ion product of water substance, 0–1000 °C, 1–10000 bars new international formulation and its background. J. Phys. Chem. Ref. Data.

[31] Wiebe R. (1941). The binary system carbon dioxide-water under pressure. Chem. Rev..

[32] Harned S.H., Raymond D.J. (1943). The ionization constant of carbonic acid in water and the solubility of carbon dioxide in water and aqueous salt solutions from 0 to 50°. J. Am. Chem. Soc..

[33] Ziegler K.J., Hanrahan J.P., Glennon J.D., Holmes J.D. (2003). Producing ‘pH switches’ in biphasic water-CO_2_ systems. J. Supercrit. Fluids.

[34] Farelas F., Galicia M., Brown B., Nesic S., Castaneda H. (2010). Evolution of dissolution processes at the interface of carbon steel corroding in a CO_2_ environment studied by EIS. Corros. Sci..

[35] Liu Z.M., Yang G.Y., Lu Y., Han B.X., Yan H.K. (1999). Phase equilibria of the CO_2_—Jiangsu crude oil system and precipitation of heavy components induced by supercritical CO_2_. J. Supercrit. Fluids.

[36] Jalal F., Mahmoud J. (2018). The physics of CO_2_ transfer during carbonated water injection into oil reservoirs: from non-equilibrium core-scale physics to field-scale implication. J. Petrol. Sci. Eng..

